# Clinical and molecular features of *FLT3* juxtamembrane domain missense mutations in acute myeloid leukaemia

**DOI:** 10.1111/jcmm.17608

**Published:** 2022-11-28

**Authors:** Christopher E. Jensen, Nathan D. Montgomery, Jonathan Galeotti, Matthew C. Foster, Joshua F. Zeidner

**Affiliations:** ^1^ Divisions of Hematology and Oncology, Department of Internal Medicine University of North Carolina School of Medicine Chapel Hill North Carolina USA; ^2^ Cecil G. Sheps Center for Health Services Research University of North Carolina Chapel Hill North Carolina USA; ^3^ Department of Pathology and Laboratory Medicine University of North Carolina School of Medicine Chapel Hill North Carolina USA; ^4^ Tempus‐RTP Durham North Carolina USA; ^5^ Lineberger Comprehensive Cancer Center University of North Carolina Chapel Hill North Carolina USA

**Keywords:** acute myeloid leukemia, FLT3, Precision medicine

FMS‐like tyrosine kinase 3 (FLT3) is a receptor tyrosine kinase involved in regulation of haematopoietic stem cell and progenitor cell proliferation and differentiation.^1^ Activating mutations in *FLT3* are found in 25%–35% of adults with acute myeloid leukaemia (AML), most commonly in‐frame internal tandem duplications (ITD) within the intracellular juxtamembrane domain (JMD).^2^ This domain, consisting of residues 572–603, serves an autoinhibitory function, in part by preventing the activation loop from unfolding into its active conformation.^3^ *FLT3*‐ITD mutations cause a gain‐of‐function phenotype with increased proliferation and protection from apoptosis^4^ and are associated with worse clinical outcomes.^5^ Most other activating *FLT3* mutations are found in the tyrosine kinase domain (TKD),^2^ though rare activating JMD deletions have also been described.^6^, ^7^

We identified three patients with rare *FLT3* JMD missense mutations (Table 1). There are limited data regarding implications of these mutations. Here, we report clinical characteristics and treatment outcomes of these individuals.

**TABLE 1:** Clinical, molecular and cytogenetic features of cases

	Patient 1	Patient 2	Patient 3
Age at diagnosis	74	41	36
*FLT3*‐JMD mutation	c.1775T>G, p.V592G, 13.0% VAF	FLT3‐ITD (c.1775 T>G, p.V592G, 10.6% VAF)	c.1771T>G, p.Y591D, 27% VAF
Other mutations	*NPM1* type A frameshift mutation (p.W288fs, 19.0% VAF) *DNMT3*A (c.2645G>A, p.R882H, 46.1% VAF) *IDH2* (c.419G>A, p.R140Q, 40.3% VAF)	*NPM1* type A frameshift mutation (p.W288fs, 35.4% VAF) *DNMT3A* (c.2645G > A, p.R882H, 26.3% VAF)	*PTPN11* (c.226G>A, p.E76K, 7% VAF)
Cytogenetics	Normal karyotype; FISH with trisomy 8	Normal karyotype	46XY; t(9;11)(p21.3;q23.3)
WBC count (10^9^/L) on presentation	12.2	6.9	43
Peripheral blasts (%) on presentation	Not available	5%	77%
Bone marrow blasts (%)	7%^a^	49%	89%
First‐line treatment	Azacitidine	7 + 3 & midostaurin induction. HiDAC & midostaurin consolidation × 3 cycles. MUD AlloHSCT with RIC (Fludarabine/Melphalan)	7 + 3 with high‐dose Daunorubicin induction. HiDAC consolidation. Related donor AlloHSCT with MAC (Busulfan/Fludarabine)
Response	MRD+ CR. Progression following cycle 7	MRD+ CR following induction. MRD‐ CR following consolidation	MRD‐ CR following induction
Second‐line treatment	Enasidenib		
Response	Differentiation syndrome; Stroke prior to response assessment		

Abbreviations: AlloHSCT, allogeneic haematopoietic stem cell transplantation; CR, complete response; FISH, fluorescence in situ hybridization; HiDAC, high‐dose cytarabine; JMD, Juxtamembrane domain; MUD, matched unrelated donor; MRD, minimal residual disease; WBC, white blood cell.

^a^ Blasts + promylocytes.

## 1 PATIENT 1

A 74‐year‐old woman with prior breast cancer managed with lumpectomy, chemotherapy, radiation and endocrine therapy developed erythematous, lower‐extremity‐predominant skin lesions 4 years after treatment. Biopsy of a representative lesion demonstrated myeloid leukaemia cutis. Bone marrow (BM) biopsy revealed 39% monocytes with aberrant CD56 expression, 7% blasts plus promonocytes and no increase in CD34 or CD117‐positive cells, consistent with therapy‐related myeloid neoplasm. Karyotype was normal, though FISH revealed trisomy 8 in 2.5%–3.0% of cells. Given concomitant leukaemia cutis, she was diagnosed with AML. A next‐generation‐sequencing (NGS) myeloid mutation panel (siParadigm Diagnostic Informatics) identified a type A *NPM1* frameshift mutation (p.W288fs, 19.0% variant allele frequency) as well as missense mutations in *DNMT3*A (c.2645G>A, p.R882H, VAF: 46.1%), *IDH2* (c.419G>A, p.R140Q, VAF: 40.3%) and the *FLT3* JMD (c.1775T>G, p.V592G, VAF: 13.0%).

She received Azacitidine 75 mg/m^2^ days 1–7 of 28‐day cycle, resulting in improved skin lesions and a morphologic complete response (CR). NPM1 PCR minimal residual disease (MRD) testing was positive at 1.46% NPM1/ABL1. Following cycle 7, she experienced biopsy‐confirmed recurrent leukaemia cutis. Enasidenib 100 mg daily was initiated. Her course was complicated by marked leukocytosis, worsening renal function and declining performance status despite improvement in skin lesions, prompting admission and management with steroids and hydroxyurea for differentiation syndrome. She presented to clinic 2 weeks after discharge with new unilateral neurologic symptoms concerning for stroke, and her family elected to pursue comfort‐oriented measures.

## 2 PATIENT 2

A 41‐year‐old woman presented with 6 months of dyspnea and weight loss. Evaluation was notable for profound anaemia with haemoglobin of 2.7 g/dl and white blood cell count (WBC) of 6.9 × 10^9^/L with 5% circulating blasts. Peripheral blood flow cytometry demonstrated an immunophenotype consistent with AML (moderate CD45, CD13, CD33, CD117, HLA‐DR expression; partial CD64 and CD4 expression; CD34 negative), and BM biopsy was hypercellular with 49% myeloid blasts. Routine cytogenetics were normal. In‐house NGS myeloid mutation panel revealed *FLT3‐*ITD, a type A *NPM1* frameshift mutation (VAF: 35.4%) and missense mutations in *DNMT3A* (c.2645G>A, p.R882H, VAF: 26.3%) and the *FLT3* JMD (c.1775T>G, p.V592G, VAF: 10.6%).

She received induction therapy with 7 + 3 (cytarabine & daunorubicin 60 mg/m^2^) and, given the *FLT3*‐ITD variant, midostaurin beginning on day 8. Day 21 BM biopsy revealed residual leukaemia, so she received a repeat induction course. Recovery BM biopsy demonstrated morphologic CR with negative flow cytometric MRD (HematoLogics); however, NPM1 PCR was positive at 0.06% NPM1/ABL1. Consolidation consisted of three cycles of high‐dose cytarabine (3 g/m^2^ IV Q12hours on days 1, 3 and 5) and midostaurin on days 8–21. Repeat BM biopsy showed ongoing CR without NPM1 transcripts (MRD‐negative), and the patient underwent matched unrelated donor allogeneic stem cell transplantation (ASCT). She was planned for maintenance FLT3 inhibition, though this was ultimately not initiated due to insurance difficulties. Post‐transplant course was complicated by skin and hepatic graft‐versus‐host disease (GVHD), requiring a prolonged steroid taper. She remains in MRD‐negative CR 4 years from transplant.

## 3 PATIENT 3

A 36‐year‐old man developed oral pain and cervical lymphadenopathy, managed with amoxicillin for possible dental infection. Due to worsening symptoms, he presented to an emergency department, where evaluation was notable for WBC 43.0 × 10^9^/L with 77% blasts and platelets of 71 × 10^9^/L. Peripheral blood flow cytometry demonstrated an immature cell population with moderate CD45 expression and co‐expression of CD4, CD11b, CD11c, CD15, CD33, CD38, CD56, CD64, CD71, CD117 and HLA‐DR, consistent with aberrant myeloid blasts. BM biopsy was markedly hypercellular with 89% myeloid blasts. Cytogenetic studies revealed t(9;11)(p21.3;q23.3) resulting in *KMT2A::MLLT3* fusion. In‐house NGS myeloid mutation panel demonstrated missense mutations in *FLT3* JMD (c.1771T>G, p.Y591D, VAF: 27%) and *PTPN11* (c.226G>A, p.E76K, VAF: 7%).

He received induction with 7 + 3 (cytarabine & daunorubicin 90 mg/m^2^). Recovery BM biopsy demonstrated morphologic CR with negative MRD by flow cytometry (HematoLogics). He received high‐dose cytarabine consolidation for four cycles, followed by related donor ASCT. Post‐transplant course was complicated by hepatic GVHD, requiring a prolonged tacrolimus and steroid taper. He remains in MRD‐negative CR at 18 months from transplant.

## 4 DISCUSSION

Previously described JMD missense mutations are summarized in Table 2. Their prevalence varies in these series, ranging from 0.4% to 2.1%, though use of older diagnostic techniques may contribute to incomplete case ascertainment in earlier cohorts. In the Catalogue of Somatic Mutations in Cancer (COSMIC), JMD missense mutations account for 0.39% of *FLT3* variants among AML samples (63 such variants among 68,507 samples),^8^ including single prior descriptions of V592G^9^ and Y591D.^5^

**TABLE 2:** Previously reported rare FLT3‐JMD missense mutations in AML

*FLT3*‐JMD variant	References
V579A	Stirewalt et al.^13^
V592A	
F590G/Y591D	
Y591C	Reindl et al.^12^
V592A	
F594L	
Y572C	Frohling et al.^9^
V592G	
S574G^a^	Syampurnawati et al.^14^
E598G^a^	
F594I^b^	Gianfelici et al.^10^
L576Q^c^	Martinez‐Lopez et al.^11^
V592A^a^	Janke et al.^4^

^a^ Concurrent FLT3‐ITD mutations.

^b^ Cytogenetics: 47XY,t(9;11)(p22;q23),+8.

^c^ Cytogenetics: complex karyotype including −7, +8.

Data regarding clinical characteristics of these patients are particularly limited. Gianfelici et al.^10^ describe a 78‐year‐old individual presenting with hyperleukocytosis who died before treatment could be initiated. Martinez‐Lopez et al.^11^ report a 68‐year‐old man with AML who progressed through or relapsed following four lines of therapy, prompting compassionate use of sorafenib, with subsequent clearance of peripheral blasts for 5 months before fatal relapse. The *FLT3* JMD missense mutation was persistently detected at the time of sorafenib initiation, though the variant could not be detected at the time of final relapse.

Prior in vitro data support the oncogenic potential of JMD missense mutations. Reindl and colleagues developed models of V579A, V592A, F594L, and F590G/Y591D *FLT3* variants via site‐directed mutagenesis in BaF3 cells, a murine interleukin‐3 (IL‐3) dependent haematopoietic cell line.^12^ Affected cells exhibited increased IL‐3‐independent growth, growth in response to FLT3 ligand, resistance to apoptosis upon IL‐3 withdrawal and FLT3 autophosphorylation compared with *FLT3*‐wildtype controls. Each of these findings was less pronounced among the *FLT3* JMD missense mutants than in ITD‐ or TKD‐expressing cell lines, suggesting that *FLT3* JMD missense mutations result in more modest gain of function. Introduction of the FLT3 inhibitor midostaurin resulted in growth arrest among the JMD missense mutant cell lines. Frohling and colleagues subsequently reported similar IL‐3‐independent growth for Y572C and V592G‐expressing BaF3 cells, along with inhibition by midostaurin.^9^ Similar data regarding the utility of pharmacologically targeting rare activating *FLT3* JMD deletions have recently been published.^6^

*FLT3* JMD missense mutations may have therapeutic implications. Midostaurin inhibits pro‐proliferative and antiapoptotic signalling associated with *FLT3* JMD missense mutations,^9^, ^12^ and at least some clinical antileukemic activity has been reported.^11^ These variants may be under‐reported given limitations of older diagnostic techniques and their unclear clinical implications to date. Additional studies in the context of modern molecular diagnostic techniques may provide further insight into the prevalence and relevance of these mutations, as well as the genomic interplay of these mutations with other abnormalities.

## AUTHOR CONTRIBUTIONS

**Christopher E. Jensen:** Conceptualization (supporting); methodology (equal); writing – original draft (lead). **Nathan D. Montgomery:** Data curation (equal); resources (equal); writing – review and editing (equal). **Jonathan Galeotti:** Data curation (equal); writing – review and editing (equal). **Matthew C. Foster:** Conceptualization (equal); writing – review and editing (equal). **Joshua F. Zeidner:** Conceptualization (lead); methodology (equal); writing – review and editing (equal).

## CONFLICT OF INTEREST

The authors report not competing interests with the present work. NDM is currently an employee of Tempus and has previously served as an advisor to Premier, Inc. MCF has received research funding from Bellicum Pharmaceuticals, Macrogenics and Rafael Pharmaceuticals and has served as advisor/consultant to Macrogenics, Daiichi Sankyo and Agios. JFZ has served as an advisor/consultant to AbbVie, Bristol Myers Squibb, Genentech, Gilead, Immunogen, Servier, Shattuck Labs and has received research funding from AbbVie, Arog, Astex, Gilead, Merck, Stemline, Syndax and Takeda. This research was partially supported by a National Research Service Award Pre‐Doctoral/Post‐Doctoral Traineeship from the Agency for Healthcare Research and Quality sponsored by The Cecil G. Sheps Center for Health Services Research, The University of North Carolina at Chapel Hill, Grant No. T32‐HS000032.

## Data Availability

The data that support the findings of this study are available on request from the corresponding author. The data are not publicly available due to privacy or ethical restrictions.
